# Role of Hemoperfusion With CytoSorb Associated With Continuous Kidney Replacement Therapy on Renal Outcome in Critically III Children With Septic Shock

**DOI:** 10.3389/fped.2021.718049

**Published:** 2021-08-24

**Authors:** Gabriella Bottari, Giulia Lorenzetti, Flavia Severini, Andrea Cappoli, Corrado Cecchetti, Isabella Guzzo

**Affiliations:** ^1^Pediatric Emergency Department, Pediatric Intensive Care Unit, Bambino Gesù Children's Hospital, IRCCS, Rome, Italy; ^2^Department of Pediatrics, University of Rome Tor Vergata, Residency School of Pediatrics, Bambino Gesù Children's Hospital, IRCCS, Rome, Italy; ^3^Department of Pediatrics, Division of Nephrology and Dialysis, Bambino Gesù Children's Hospital, IRCCS, Rome, Italy

**Keywords:** CytoSorb, hemoperfusion, blood purification, children, PICU, acute kidney injury, septic shock

## Abstract

**Introduction:** Sepsis-associated acute kidney injury (SA-AKI) represents a relevant cause of mortality and morbidity in critically ill children. Since with the “inflammatory theory” the authors have been witnessed an important role of inflammatory mediators in the pathophysiology and in the prognosis of SA-AKI, making the need of adjunctive therapies in association with kidney replacement therapies mandatory. Hemoperfusion with CytoSorb is a safe and well-tolerated therapy in septic shock: the very high surface area of the absorber means it is able to efficiently remove cytokines and other medium size molecules involved in cytokine storm, thus playing a synergistic effect with Continuous Kidney Replacement Therapy (CKRT).

**Materials and Methods:** We retrospectively analyzed data from a cohort of eight critically ill children treated from January 2018 to March 2020 describing the impact of CKRT plus hemoperfusion with CytoSorb on renal outcome in critically ill children with septic shock.

**Results:** We evidenced a significant reduction in interleukin (IL)-6 an IL-10 after hemoperfusion with CytoSorb in our pediatric population. Furthermore, we were able to show a significant improvement of creatinine and blood urea nitrogen (BUN) after blood purification and at pediatric intensive care units (PICU) discharge. We have observed a median of 2.5 CKRT days after stop of hemoperfusion (Q_1_ 0.25; Q_3_ 18.75). None of our patients required CKRT 30 days after PICU discharge (PICU-D). None of them developed CKD.

**Conclusion:** Hemoperfusion with CytoSorb is a valuable therapeutic option in combination with CKRT in SA-AKI. More studies are warranted to confirm our results and in particular to define the role of this adjuvant therapy as a preemptive strategy to protect renal function in pediatric septic shock.

## Introduction

Sepsis-associated acute kidney injury (SA-AKI) refers to impairment of renal function in sepsis and septic shock. In the pediatric setting, a large multicenter study reported that AKI developed in 26.9% and severe AKI in 11.6% of patients admitted to pediatric intensive care units (PICU) (1). Furthermore, septic children with AKI had a higher 28-day mortality than septic children without AKI (11 vs. 2.5%) (1). Severity of AKI affects length of stay in PICU/hospital, duration of mechanical ventilation, kidney replacement therapy (KRT), and extracorporeal membrane oxygenation (ECMO) (2) and confers a higher risk of developing chronic kidney disease (CDK) in the 5 years after discharge (3). Because of its specific physiopathology, SA-AKI is considered a specific subtype of AKI (4). Sepsis induces renal hypoperfusion and changes in the intrarenal microvasculature leading to a reduction of the glomerular filtration rate (5). Molecules from the pathogens and cytokines [such as interleukin (IL)-6, IL-8, IL-18, tumor necrosis factor (TNF)-α] interact with tubular and endothelial cells, causing multiple biological alterations: loss of cell polarity, dysfunction of the basolateral compartment, apoptosis (4, 6), and even cell cycle arrest (7, 8). This reprogramming of renal cell metabolism in response to inflammation seems to be directed to lower energy consumption as a defense mechanism and could be involved in the progression to CKD (9). Interestingly, histological studies on septic patients did not evidence significant tubular apoptosis or necrosis when compared to other types of AKI (10), and the structural damage was limited to few areas and did not correlate with duration of AKI (11). This means that AKI causes cellular dysfunction rather than death and that prevention and early treatment of AKI may be able to reverse cellular dysfunction (12). In this context, the need of adjunctive therapies is mandatory and one of the most promising new strategies is the modulation of the host response by the removal of the inflammatory mediators and/or bacterial toxins through extracorporeal blood purification therapies (EBPTs) (13).

**Figure 1:**
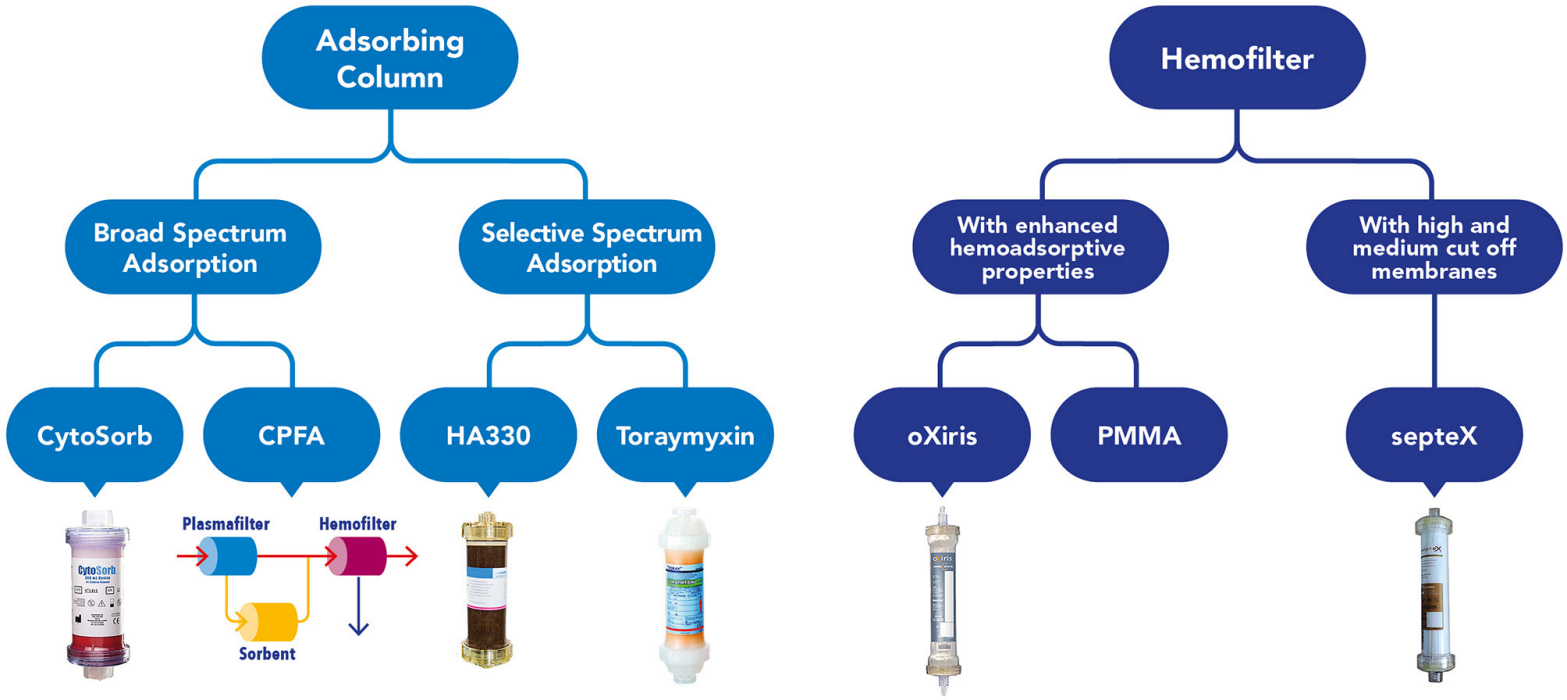
Graphical overview of the current available devices to perform blood purification in critically ill children affected by septic shock.

It has been demonstrated that continuous kidney replacement therapy (CKRT) filters can impact on cytokines removal firstly by adsorption and this ability is more evident in the first hours of the hemofilter life (14). The ability to remove cytokines by convection are limited by a hemofilter's membrane cut off (35 kD). Furthermore, we have considered the described decay effect on the Sieving coefficient (a progressive reduction of the pore size of the membrane along the life of a hemofilter) (14, 15). Therefore, during the years multiple adjunctive techniques have been developed such as hemoperfusion, high-volume hemofiltration, high cut-off membrane hemofiltration/hemodialysis, plasma separation techniques, immunoglobulin therapy, endotoxin-binding polymyxin B hemoperfusion. Some of them have shown good results in terms of hemodynamic stability, but their use is not standardized due to lack of randomized control trials (RCTs) (16) (Figure 1). Among them, hemadsorption techniques allow the removal of molecules which could not be removed from circulation with traditional filters, through the direct contact of blood with adsorbent surfaces via an extracorporeal circuit. The CytoSorb technology is a hemoperfusion sorbent cartridge characterized by a resin with polymer beads that allows the absorption of pro- and anti-inflammatory cytokines through a combination of size exclusion and hydrophobic interactions (17). *In vitro* experiments demonstrated that CytoSorb efficiently removes a broad spectrum of inflammatory mediators including toxic pathogen associated molecular patterns (PAMPS) and damage associated molecular patterns (DAMPS), cytokines, and mycotoxins from blood (18). Previous *in vivo* studies showed that CytoSorb is a safe and well-tolerated rescue therapy in adult and pediatric population with severe septic shock, leading to more favorable clinical outcome when initiated early [within 24–48 h after onset of septic shock (19–21)].

To our knowledge, few published studies focused on SA-AKI and renal outcome in septic patients who have received hemadsorption with CytoSorb, and none of them included pediatric patients (22). Therefore, the aim of this retrospective study was to evaluate the clinical and renal outcome after CytoSorb in association to CKRT in pediatric patients with sepsis or septic shock admitted to PICU.

## Materials and Methods

**Figure 2:**
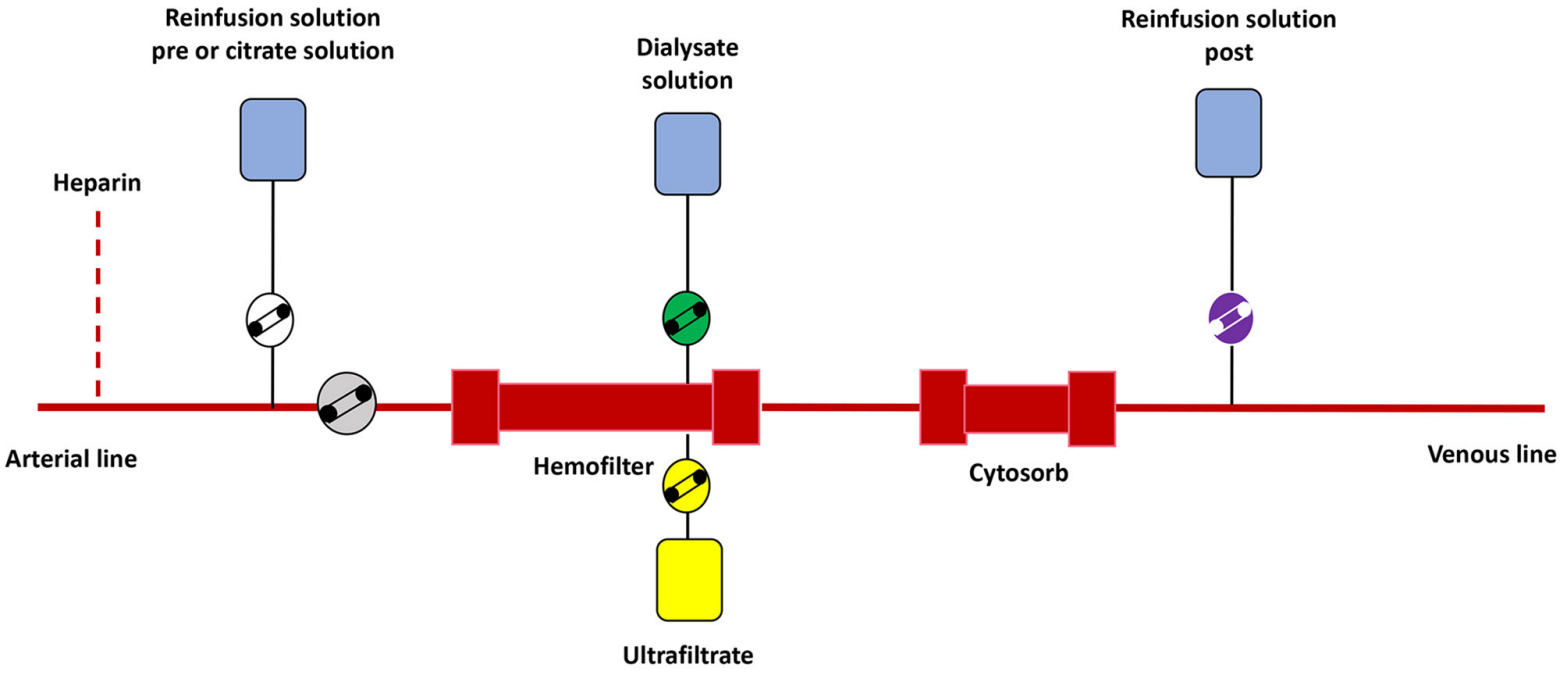
Graphical schema of the extracorporeal blood purification circuit.

This study has been acknowledged by the Ethical Committee of Children Hospital Bambino Gesù. We retrospectively analyzed data of a cohort of eight critically ill children treated from January 2018 to March 2020 describing the impact of CKRT plus hemoperfusion with CytoSorb on acute kidney injury associated to septic shock. Hemoperfusion has been performed with CytoSorb, a cartridge containing biocompatible polystyrene divinylbenzene copolymer beads capable of adsorbing molecules of medium molecular weight using a combination of size exclusion and hydrophobic interactions. The priming volume of the cartridge is 120 ml. Continuous Kidney Replacement Therapy was performed with prismaflex monitor and standard hemofilter [Polyarylethersulphone or AN69 heparin coated filter (ANST69), 0.2–0.6–1 m^2^ according to body weight, blood volume 60, 93, and 152 ml, respectively], combined with the adsorber in continuous veno-venous hemofiltration (CVVH) or continuous veno-venous hemodiafiltration (CVVHDF) modality, using a pre-/post-filter reinfusion and an effluent dose of 2,000 ml/h/1.73 m^2^. The cartridge was inserted in the CKRT circuit in series with the hemofilter in a post-filter position (Figure 2); both CKRT circuit and adsorber were flushed with saline solutions and primed with albumin, blood, or normal saline at discretion of the attending physicians. A hemodialysis catheter was inserted into a central vein (internal jugular or femoral) as appropriate according to children size. Anticoagulation was managed with a continuous infusion of unfractionated heparin (UFH) sodium (10–20 UI/kg/h) to achieve a post-filter activated clotting time (ACT) between 160 and 180 s. In case of contraindications to UFH, regional citrate anticoagulation was used. Hemoperfusion was continued on the basis of the clinical course as well as laboratory biomarkers (ferritin, C-reactive protein, metabolic status including pH, inotropic, and vasopressor load). The hemofilter was changed every 72 h and the absorber was changed every 24 h as recommended by the manufacturer and continued for a maximum of 96 h (mean 72 h). In patients on extracorporeal ECMO, the CKRT access was placed post-oxygenator and the CKRT return post-pump. Septic shock was treated following the latest guidelines of the Surviving Sepsis Campaign (23). Acute kidney injury and its stage of severity have been defined according to the Kidney Disease: Improving Global Outcomes (KDIGO) criteria (24) and indications for CKRT were fluid overload, electrolyte imbalance or both. Hemoperfusion treatment with CytoSorb was performed as a rescue therapy within 24 h after the proved or suspected diagnosis of septic shock in case of an inadequate response to standard therapy (defined by an increase of lactate concentrations and/or an increased vasopressor requirements or additional need of inotropes) after an observational period of a maximum of 6 h or immediately in parallel to standard therapy in patients with refractory septic shock. To assess the therapeutic impact of the combined CKRT-hemoperfusion treatment we measured IL-6 and IL-10 immediately before the start (T0) and at the end of the hemoperfusion treatment (T1). For each patient we described treatment modalities (ventilation days, CKRT days, ECMO days when required) and outcomes (PICU stay, Hospital stay, PICU mortality, 28-Day mortality, Hospital mortality). Continuous data were represented as median and interquartile range. Data at different time (pre- and post-treatment) were compared trough Wilcoxon signed-rank test. Differences were considered statistically significant at *p* < 0.05. All statistical analyses were performed using Stata 13.1.

## Results

**Figure 3:**
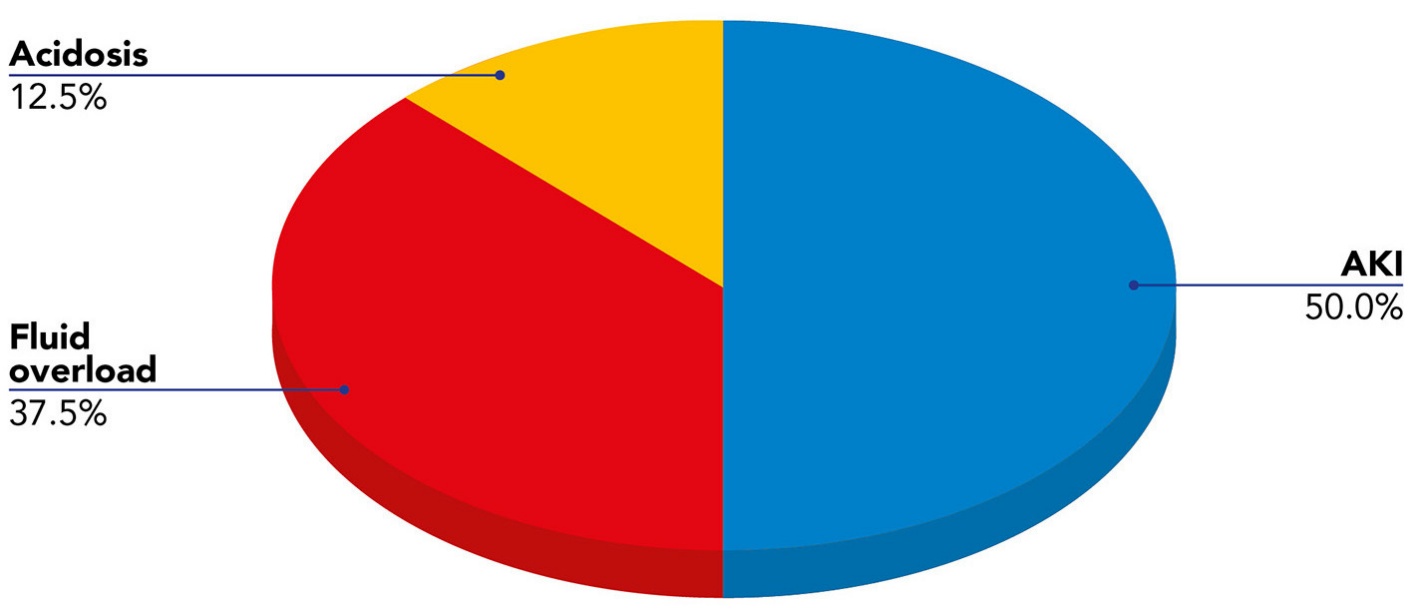
Indications for blood purification (CKRT plus hemoperfusion) in our cohort of pediatric patients with septic shock.

We present data of eight critically ill children with septic shock. Patients' characteristics at PICU admission and at start of blood purification (sex; age; weight; source of infection; comorbidity; etiology of infection) are reported in Table 1. The main reason for CKRT plus hemoperfusion in our cohort is depicted (AKI 50%; Fluid overload 37.5%; Acidosis 12.5%) in Figure 3. Continuous veno-venous hemofiltration was the preferred modality in one patient whereas seven patients were treated with CVVHDF. We have calculated the median values and quartiles (Q_1_,Q_3_) before (T0) and after (T1) hemoperfusion of creatinine (mg/dl) (T0 median 0.74 [Q_1_ 0.42, Q_3_ 1.40]; T1 median 0.31 [Q_1_ 0.2, Q_3_ 0.41]), blood urea nitrogen (BUN) (mg/dl) (T0 median 22.5 [Q_1_ 13.7, Q_3_ 32.7]; T1 median 16.5 [Q_1_ 9.7, Q_3_ 30.5]); lactate (mmol/L) (T0 median 7.05 [Q_1_ 3.37, Q_3_ 13.5]; T1 median 1.3 [Q_1_ 0.9, Q_3_ 5.8]); urinary output (ml/kg/h) (T0 median 2.13 [Q_1_ 0.14, Q_3_ 4.2]; T1 median 1.38 [Q_1_ 0.04, Q_3_ 3.6]); IL-6 (pg/ml) (T0 median 1801.27 [Q_1_ 173.3, Q_3_ 19.33]; T1 median 203.1 [Q_1_ 46.9, Q_3_ 1583.72]); IL-10 (pg/ml) (T0 median 178.78 [Q_1_ 115.39, Q_3_ 414.13]; T1 median 22.68 [Q_1_ 6.85, Q_3_ 84.73]).

**Table 1:** Patients' characteristics and details on the primary source of sepsis, comorbidity, etiology of infection.

**ID**	**Sex**	**Age**	**Weight (kg)**	**Primary source of septic shock**	**Comorbidity**	**Etiology of infection**	**PIM-3 (%)**
1	M	17 m	12	Gastrointestinal infection	Typical HUS	*Escherichia coli* STEC O26	28
2	F	12 m	9	Septic shock	f-HLH	*Klebsiella pneumoniae*	87
3	F	5 y	26	Septic shock, myocardial disfunction	_	Epstein-Barr virus	23
4	F	12 d	3.130	Septic shock, pneumonia	_	*Serratia marcescens*	21
5	M	5 y	20	Abdominal sepsis, hypovolemic shock	Bowel malrotation	*Hemophilus influenza, Streptococcus pyogenes*	2.5
6	F	14 y	45	Bacteremia	Cystic fibrosis with pancreatic insufficiency	Methicillin-resistant *Staphylococcus aureus*	23
7	M	11 d	3.600	Abdominal sepsis	Right diaphragmatic hernia	*Serratia marcescens, Pseudomonas aeruginosa*	18
8	F	5 y	18	Viral myocarditis	Secondary HLH, MODY2 diabetes	Human Herpes virus-6	12

*M male; F, female; y, years; d, days; m, months; HUS, hemolytic uremic syndrome; HLH, hemophagocytic lymphohistiocytosis; STEC, shiga toxin-producing Escherichia coli. PIM-3 Pediatric Index Mortality 3 at PICU admission*.

**Figure 4:**
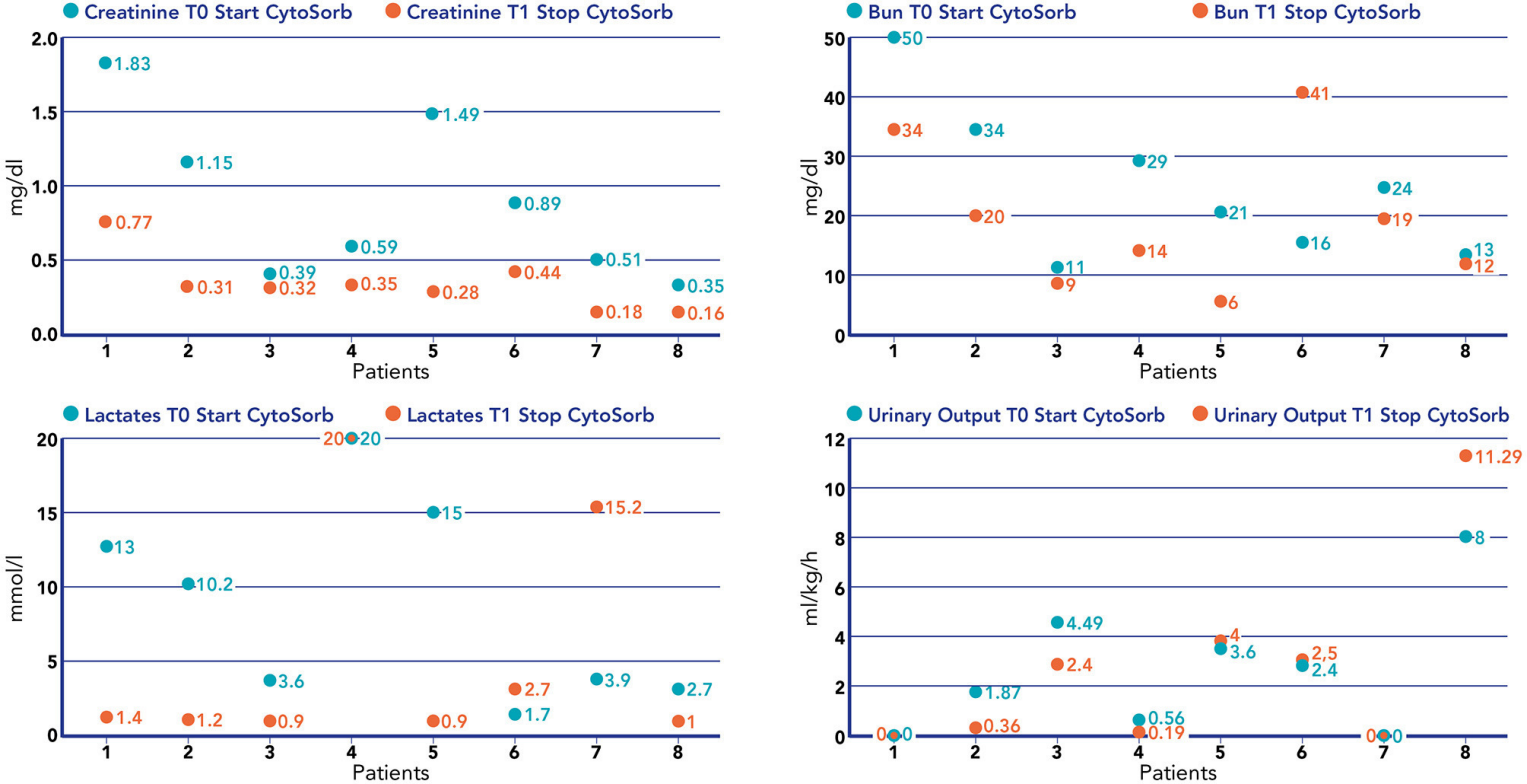
Values of creatinine (mg/dl), BUN (mg/dl), lactate (mmol/L), and urinary output (mL/kg/h) before the start of hemoperfusion with CytoSorb and after the stop of hemoperfusion with CytoSorb in each patient treated.

**Figure 5:**
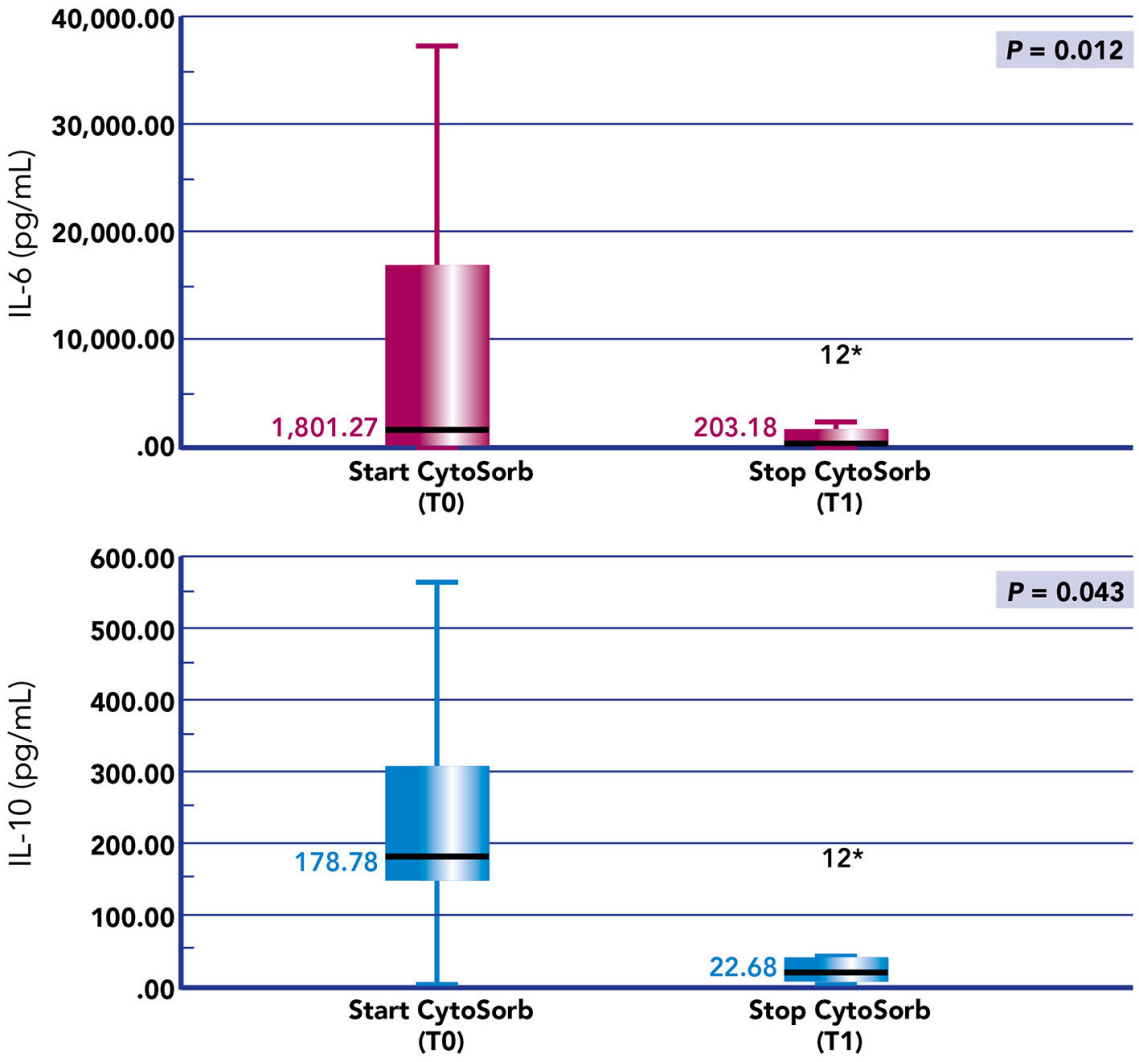
Box plots of IL-6 and IL-10 before and after hemoperfusion with CytoSorb in our cohort of pediatric patients with septic shock. *p-value < 0.05.

**Figure 6:**
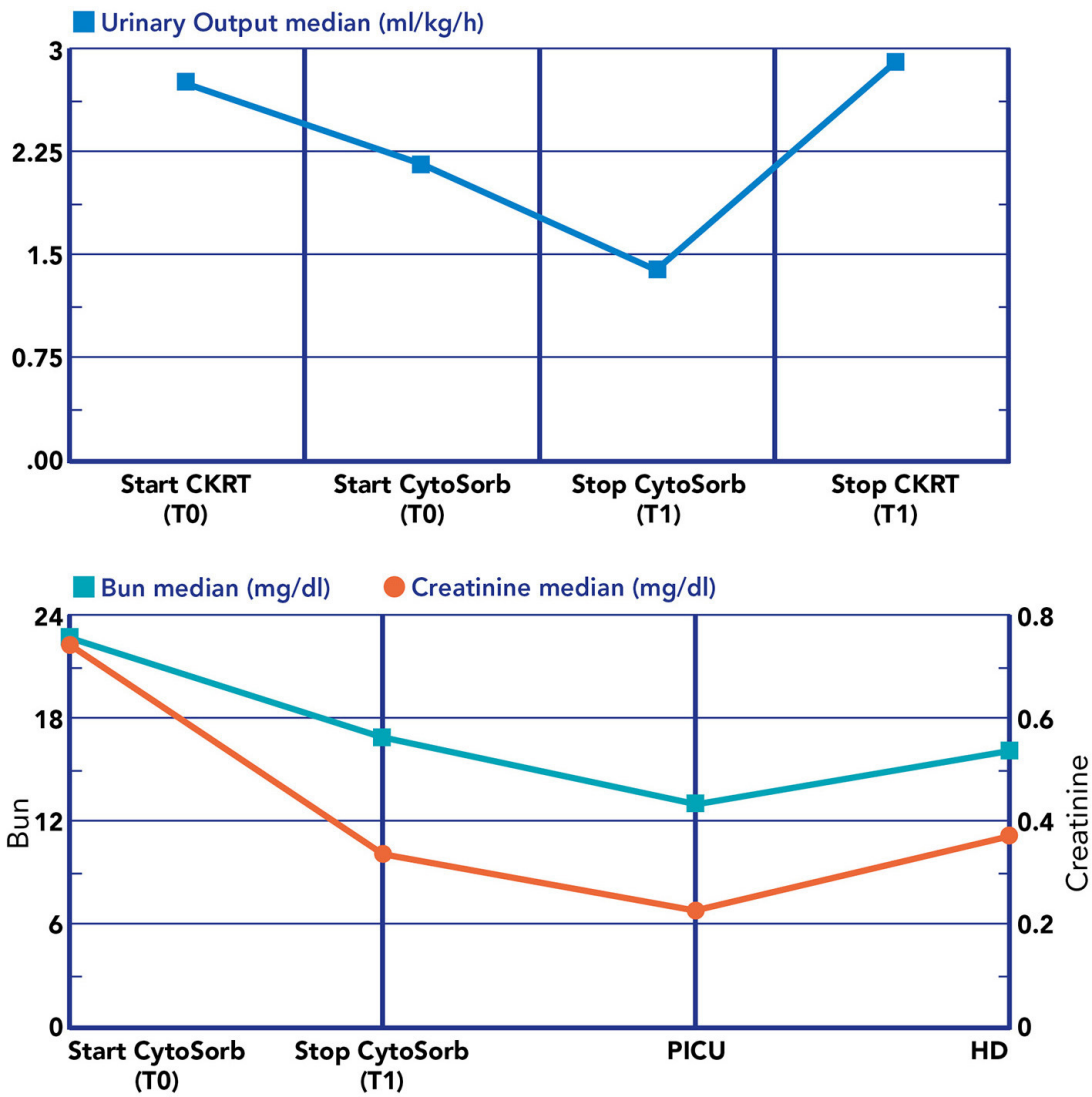
Renal biomarkers and urine output during the blood purification journey. **Lower section**: T0, immediately before the start of CRRT plus hemoperfusion with CytoSorb; T1, after the stop hemoperfusion with CytoSorb; PICU, at PICU discharge; HD, at hospital discharge.

We noticed a statistical difference (*p* < 0.05) before (T0) and after (T1) hemoperfusion for creatinine (*p* = 0.012); IL-6 (*p* = 0.012); IL-10 (*p* = 0.043) (Table 2) (Figure 4). Furthermore, we have described the renal outcome in our cohort through monitoring the median of creatinine, BUN, and urinary output. Median values were calculated at the most significant clinical time points of the blood purification journey (Figure 5) represented by start of CKRT, start of hemoperfusion, stop of hemoperfusion, PICU discharge (PICU-D) and hospital discharge (HD). Blood urea nitrogen (mg/dl) and creatinine (mg/dl) at PICU-D and creatinine at HD showed a significant reduction by the start of the blood purification. Creatinine PICU-D median 0.215 (Q_1_ 0.18, Q_3_ 0.34) (*p* = 0.017). Creatinine HD median 0.35 (Q_1_ 0.20, Q_3_ 0.53) (*p* = 0.050). BUN PICU median 12.5 (Q_1_ 12, Q_3_ 14.75) (*p* = 0.021). Figure 6 describes the values of creatinine, BUN, urinary output, and lactate before and after hemoperfusion in each patients treated.

**Table 2:** Statistical evaluation of renal outcome parameters before (T0) and after (T1) hemoperfusion with CytoSorb.

	**T0**	**T1**	**Median difference (T0–T1)**	***p***
	**Median (Q1–Q3)**	**Median (Q1–Q3)**		
Creatinine (mg/dl)	0.74 (0.425–1.405)	0.315 (0.205–0.418)	0.425	^*^0.012
BUN (mg/dl)	22.5 (13.75–32.75)	16.5 (9.75–30.5)	6	0.161
Urinary output (ml/kg/h)	2.135 (0.14–4.268)	1.38 (0.048–3.625)	0.755	0.917
Lactate (mmol/L)	7.05 (3.37–13.5)	5.75 (0.9–58)	2.35	0.282
IL-6 (pg/ml)	1801.27 (173.3–19333.445)	203.175 (46.91–1583.725)	1598.095	^*^0.012
IL-10 (pg/ml)	178.78 (115.39–414.13)	22.68 (6.853–84.735)	156.1	^*^0.043
Hb (g/dl)	10.75 (8.525–12.225)	10.05 (9.475–10.95)	0.7	0.833
PLT (/mcl)	129,000 (21,250–393,000)	150,500 (46,250–239,250)	−21,500	0.263

*Numerical data in bold indicates significant value with p < 0.05. BUN, blood urea nitrogen; IL, interleukin. Data are shown as median [interquartile range (IQR)], IQR: Q1–Q3.*

* *p-value < 0.05: Wilcoxon Rank-Sum Test. Hemoglobin (Hb); Platelets (PLT)*.

The need for diuretics after the ending of CKRT has been observed in three out of eight patients (37.5%) and the need for anti-hypertensive drugs at HD has been noticed in three out of eight patients (37.5%). We have observed a median of 2.5 CKRT days after stop of hemoperfusion (Q_1_ 0.25, Q_3_ 18.75). None of our patients required CKRT 30 days after the PICU-D.

We reported the most important clinical outcomes in our cohort expressed as PICU days, PICU mortality, 28-day mortality, hospital mortality, hospital length of stay, and we have related them to the type of extracorporeal support received (cycles of hemoperfusion; CKRT days; need of ECMO) (Table 3). Three out of eight patients (37.5%) in our cohort did not survive to PICU-D.

**Table 3:** Treatment modalities and patients' clinical outcome.

**ID**	**CytoSorb treatments (*n*)**	**CKRT (days)**	**ECMO**	**PICU stay (days)**	**PICU mortality**	**28-Day mortality**	**Hospital stay (days)**	**Hospital mortality**
1	4	34	VA	58	No	No	74	No
2	3	21	_	22	Yes	Yes	123	Yes
3	3	5	_	11	No	No	28	No
4	1	1	VA	16	Yes	Yes	16	Yes
5	3	5	_	13	No	No	77	No
6	3	22	VV	19	No	No	121	No
7	2	43	VA	43	Yes	Yes	43	Yes
8	3	6	VA	13	No	No	47	No

*ECMO, extracorporeal membrane oxygenation; CKRT, continuous renal replacement therapy; PICU, pediatric intensive care unit; VA, veno-arterial; VV, veno-venous*.

## Discussion

It has been recognized that kidney involvement in septic shock is not only related to intrarenal hemodynamic dysfunction, but is also a consequence of the dangerous effects of medium size molecules (cytokines, chemokines, complement fragments, and the like) which have a toxic effect on tubular cells when concentrated in the ultrafiltrate, acting on the luminal surface of the tubules (12). For these reasons CKRT conventional indications in SA-AKI (refractory acidosis, hyperkalemia, uremia, oliguria-anuria, fluid overload unresponsive to diuretics) have been expanded to KRT technique which focus also on blood purification (25). This concept has been reinforced since it was shown that these “toxins” in septic shock induce a detrimental organ crosstalk causing hepatorenal and cardiorenal syndromes and the production of nephrotoxic molecules from the lungs, brain and bone marrow which can worsen the multiple organ dysfunction pathophysiology (11). Recently, Brouwer et al. demonstrated that CytoSorb hemoperfusion associated with CKRT reduces the 28-days all-cause respect to CKRT alone and this effect seem to be more relevant in patients with a high organ disease score and high level of lactates (26). These data point the attention to the potential advances of kidney replacement techniques for improving morbidity and mortality in septic shock. We report a retrospective analysis on a small cohort of children affected by septic shock and treated with blood purification approach based on CKRT plus hemoperfusion. To the best of our knowledge this is the first paper describing the impact of this type of adjuvant therapy on renal outcome in pediatric septic shock.

Our findings show that hemoperfusion significantly contributes to clearance of two of the most important cytokines (IL-6 and IL-10) involved in SA-AKI. We have observed a positive impact of this blood purification strategy on some important renal biomarkers: creatinine has shown a significant improvement at the stop of hemoperfusion but also at PICU and HD and the same positive variation has been observed for BUN at PICU-D. Unfortunately, we have not monitored other biomarkers of renal damage where we could more sensitively detect renal function improvements also in those patients who did not meet the full criteria for AKI. However, considering the progressive and sustained improvement in renal function in our cohort and that no one of our survived patients required CKRT at 30 days or had a clinical picture of CKD, we can postulate that together with CKRT the adjunctive action of hemoperfusion played a significant role on renal outcome in our cohort trough a massive removal of medium-sized molecules which lead toxic effect on tubular cells.

Two of three patients who did not survived were newborns confirming the high morbidity and mortality in small children with severe septic shock and with need of extracorporeal support (27, 28).

Our study provides important information on the potential role of CytoSorb hemoperfusion plus CKRT in preventing and treating SA-AKI in children with septic shock. We would outline some limitations; our cohort was very small and under-powered to draw definitive conclusion by our statistical evaluation. Some important aspects need to be elucidated with further studies, primarily the best timing to start with this type of adjunctive therapy and the potential removal of antibiotics or other useful drugs with the two devices. As it has been debated for CKRT about right time and the potential role of CKRT without AKI as a preemptive strategy to protect renal function and gain hemodynamic stability, the same concept should be applied for hemoperfusion with CytoSorb as it has emerged that removal of inflammatory mediators from the bloodstream is thought to possibly improve clinical outcomes (29). We would also underline the synergistic action between hemoperfusion and CKRT: this latter is very helpful correcting fluid overload, electrolytic and acid-base imbalance, which is why we always combine these two techniques and the extremely high surface membrane of cartridge used for the hemoperfusion, to implement the adsorptive action of medium size molecules with a huge removal from the bloodstream and the tissue (30, 31). Finally, the role of hemoperfusion with CytoSorb has not yet investigated in small children (<10 kg) except for anecdotal case reports. Considering the characteristics of the device with high volume of priming it remains to be highlighted the potential benefits and adverse effects of these adjunctive techniques in small children.

## Conclusion

Although we acknowledge that definitive conclusions cannot be drawn from this a small cohort, we highlight that our experience shows that hemoperfusion with CytoSorb is a valuable therapeutic option in SA-AKI. The use of adsorption columns is attractive, since these devices can be combined with CKRT for the concomitant treatment of fluid overload and AKI, and at the same time, play an important role in managing cytokine storm associated with organs damage in septic shock. More evidence is required to better define the role of CytoSorb therapy in this clinical setting in the proper time of onset of this adjuvant therapy.

## Data Availability Statement

The raw data supporting the conclusions of this article will be made available by the authors, without undue reservation.

## Ethics Statement

The studies involving human participants were reviewed and approved by Ethical Committee Children Hospital Bambino Gesù. Written informed consent to participate in this study was provided by the participants' legal guardian/next of kin.

## Author Contributions

GB drafted the manuscript. GL and FS analyzed and interpreted the data and written the manuscript with GB. AC, CC, and IG discussed the results and reviewed the manuscript. All authors have read and approved the final manuscript.

## Conflict of Interest

The authors declare that the research was conducted in the absence of any commercial or financial relationships that could be construed as a potential conflict of interest.

## Publisher's Note

All claims expressed in this article are solely those of the authors and do not necessarily represent those of their affiliated organizations, or those of the publisher, the editors and the reviewers. Any product that may be evaluated in this article, or claim that may be made by its manufacturer, is not guaranteed or endorsed by the publisher.
